# Genetic Variation of MSP-1 Gene in *Plasmodium vivax* Isolated from Patients in Hormozgan Province, Iran using SSCP-PCR

**Published:** 2012

**Authors:** A Miahipour, H Keshavarz, A Heidari, A Raeisi, M Rezaeian, S Rezaie

**Affiliations:** 1 Department of Medical Parasitology and Mycology, School of Public Health, Tehran University of Medical Sciences, Tehran, Iran; 2 Center for Research of Endemic Parasites of Iran (CREPI), Tehran University of Medical Sciences, Tehran, Iran; 3 Department of Pathobiology, School of Medicine, Alborz University of Medical Sciences, Karaj, Iran; 4 Department of Medical Entomology and Vector Control, School of Public Health, Tehran University of Medical Sciences, Tehran, Iran; 5 Department of Medical Biotechnology, School of Advanced Technologies in Medicine, Tehran University of Medical Sciences, Tehran, Iran

**Keywords:** *Plasmodium vivax*, MSP-1, Polymorphism, SSCP-PCR, Iran

## Abstract

**Background:**

The main goal of present study was to detect polymorphism in MSP-1 gene which is a major blood stage candidate for vaccine in *Plasmodium vivax* by Single Strand Conformational Polymorphism-Polymerase Chain Reaction (SSCP-PCR).

**Methods:**

During 2008 to 2010 fifty samples were collected from Iranian patients with *P. vivax* in Hormozgan Province, southern Iran. All of the samples were detected by microscopical examination. Amplification of MSP-1 gene was done by PCR after DNA extraction. Single strand DNAs due to using in SSCP, was electrophoresed on polyacrylamid- Bisacrylamid gel then banding patterns were revealed by silver-staining method. Sequencing as a typing method was performed for some isolates.

**Results:**

All of the 50 isolates were positive microscopically. Totally 12 (24%) isolates showed 440 bp and 38 (76%) showed 500 bp in PCR assay. SSCP analysis revealed four banding patterns. Pattern I (10/50), Pattern II (12/50), Pattern III (27/50), and Pattern IV (1/50). The results sequencing analysis of the MSP-1 gene in 19 isolates revealed diversity in nucleotides and amino acid in Iranian *P. vivax* isolates.

**Conclusion:**

Our study confirms that the SSCP-PCR is a rapid method for detecting polymorphism in MSP-1 gene in *P. vivax*. The presence of different haplotypes in MSP-1 gene shows that several *P. vivax* strains exist in malaria endemic areas of Iran.

## Introduction

*Plasmodium vivax* is the most prevalent malaria infection and an important cause of morbidity in many endemic regions in the world, especially in Asia, Central and South America (1). Presently, 2.85 billion people globally are at risk of *P. vivax* malaria infection (2). More than 50% of all malaria cases in outside of Africa occurred by *P. vivax* (3). *Plasmodium vivax* shows emergence of resistance to the antimalarial drugs and is a major concern for the current strategies of malaria control (4). It is the predominant causative species of malaria with about 90% of the total annual reported cases of malaria in Iran (5).

Development of vaccine against *P.vivax* malaria, however, is one of the priorities for control of the disease. Efficient vaccine development will require more information about genetic diversity and parasite population (6).

Merozoite surface protein-1 is a 200 kDa polymorphic glycoprotein, this protein expressed on the surface of the *P. vivax* merozoite (7). It is a major candidate for vaccine development against asexual stages (8). The most variable region of *MSP*-1 gene is flanked by interspecies conserved block 5 and 6 (ICB 5-6) (9). Two complete gene sequences of the Brazilian Belem (Bel) and Salvador (Sal-1) strains are known (8, 10).

Single strand conformational polymorphism (SSCP), is the simplest technique for detecting unknown mutation and variation in denaturated DNA fragments with mobility shifts on polyacrylamid gels. SSCP has been used extensively in organism genetic to identify mutation within genes and is able to detect single point mutation (11). In this method the target nucleotide of interest is amplified and separated as single- strand molecules by electrophoresis (12). Single strand conformational polymorphisms technique is considered as one of the sensitive, cheap and rapid method for detecting variation in nucleotide sequence (13, 14).

The main aim of this study was demonstration of variation in MSP-1 gene of *P. vivax* isolated from Hormozgan Province, southern Iran, using SSCP-PCR, as the first research in this field.

## Materials and Methods

### Blood sample collection and study site

Totally 50 blood samples were collected from *P.vivax* infected patients in Bandar-Jask Health Center in Hormozgan Province, southern Iran from December 2008 to December 2010. All clinical isolates were diagnosed as *P. vivax* by light microscopical examination of Giemsa stained thin and thick blood smears. Two ml venous blood from each patient was collected in tube containing EDTA anticoagulant solution and stored at -20 °C until DNA extraction. This study was approved by the Ethical Committee of Tehran University of Medical Science, Tehran, Iran.

### Genomic DNA purification

Genomic DNA was extracted from 200 µl of whole blood sample by using commercial QIAamp DNA blood minikit (Qiagen, Germany), according to the manufacturer's instructions.

### PCR amplification

Amplification of the fragment between ICB5 and ICB6 from *Pv*MSP-1 was conducted using F: 5^/^ -CTGGCAATACAGTCAATGCGC-3^/^ and R: 5^/^ -CATGGCTGGCAAGTTGTTCTA-3^/^ primers ( designed for this study).

The cycling parameters for PCR were as follows: 5 min initial denaturation at 95 °C followed by 35 cycles with 1 min denaturation at 94 °C, 90 seconds annealing at 64 °C, 2 min extension at 72 °C and a final primer extension at 72 °C for 10 min.

PCR production were electrophoresed on 1% agaros gel containing ethidium bromide and visualized by UV transilluminator.

The fragment sizes of PCR products were determined using 100bp DNA molecular weight marker (Fermentase).

### Single Strand Conformational Polymorphism (SSCP) processes

SSCP analysis was conducted of the PCR amplified fragments following the protocol used by Craig & Kain (15) with some modifications. Briefly, 10 µl of PCR product were mixed with 10 µl of denaturing loading buffer containing 98% formamide, 20 mM EDTA, 0.05% bromophenol blue, and 0.05% xylene cyanol. This mixture was denatured at 95 °C for 15 min and immediately chilled in ice before loading to the SSCP gel. Fifteen µl of above samples were run through nondenaturing 8% polyacrylamid- Bisacrylamid gel containing 5% glycerol. Electrophoresis was performed on a vertical electrophoresis system with 0.05 X TBE buffer at 150V for 20 h, then the gel was stained by silver staining method according to the procedure done by Brant (16) with some modifications.

### DNA sequence

Sequence analysis was used to identify polymorphism in *Pv*MSP-1gene from 19 isolates. The obtained DNA fragments were sequenced by 3730/Bioneer 3730 sequencer (BIONEER, Korea).

Nucleotide and amino acid sequences of the *Pv*MSP-1 were aligned and compared with the following published sequence Salvador *Pv*MSP-1 (accession number AAA29735) and Belem1*Pv*MSP-1(accession number AAA63427) using CLUSTALW, DnaSP, and MEGA4 softwares.

## Result

**Fig. 1:**
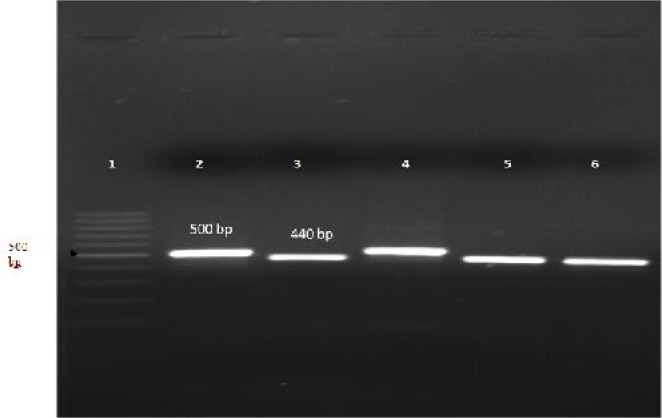
PCR products of *MSP-1* gene, Lane 1: DNA marker (ladder 100bp); lanes 3, 5 and 6: isolates type I (440 bp), lanes 2 and 4: isolates type II (500 bp)

A total of 50 blood samples were collected from patients infected with *P. vivax* in Bandar-Jask. All samples were positive for *P. vivax* by microscopical examination; no mixed infection with other malaria parasites was detected. *MSP-1* gene was amplified by PCR from parasite genomic DNA obtained from 50 isolates. On the basis of molecular weight of PCR products, the isolates could be categorized in two allelic types, including type I approximately 440 bp and type II approximately 500bp (Fig. 1).

**Fig. 2:**
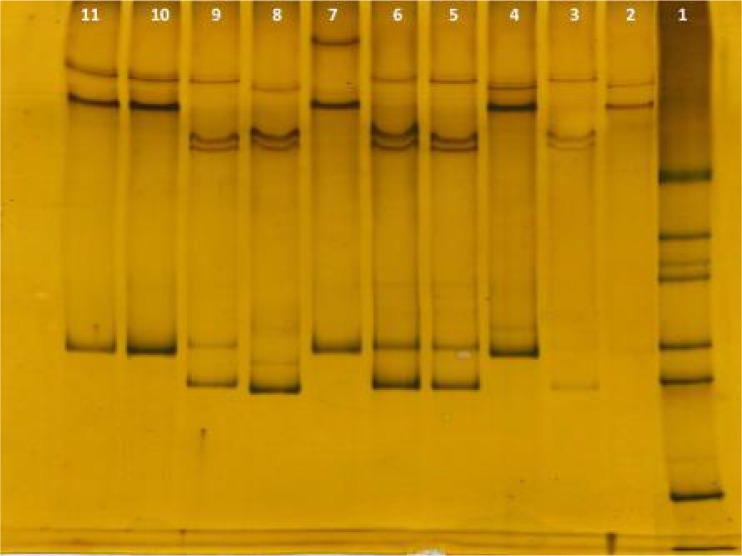
SSCP analysis for the MSP-1 gene, Lane 1: DNA ladder marker (100bp); Lane 2 and 4: Isolates with pattern I; Lane 3,5,6,8 and 9: Isolates with pattern II; Lane 10 and 11: Isolates with pattern III; Lane 7: Isolate 26 with pattern IV

Twelve (24%) isolates revealed 440 bp and 38 (76%) isolates revealed 500 bp. All PCR products from 50 patients were analyzed using single-strand conformational polymorphism (SSCP). SSCP analysis revealed four banding patterns (Fig. 2), including pattern I (10/50), pattern II (12/50), pattern III (27/50) and pattern IV (1/50).

All of the isolates with 440 bp band in PCR assay, showed pattern II in SSCP but samples with 500 bp band in PCR assay, revealed patterns I, III and IV in SSCP electrophoretic mobility. Four, six and eight isolates of patterns I, II and III were sequenced respectively. The only sample of pattern IV was also sequenced.

**Fig. 3:**
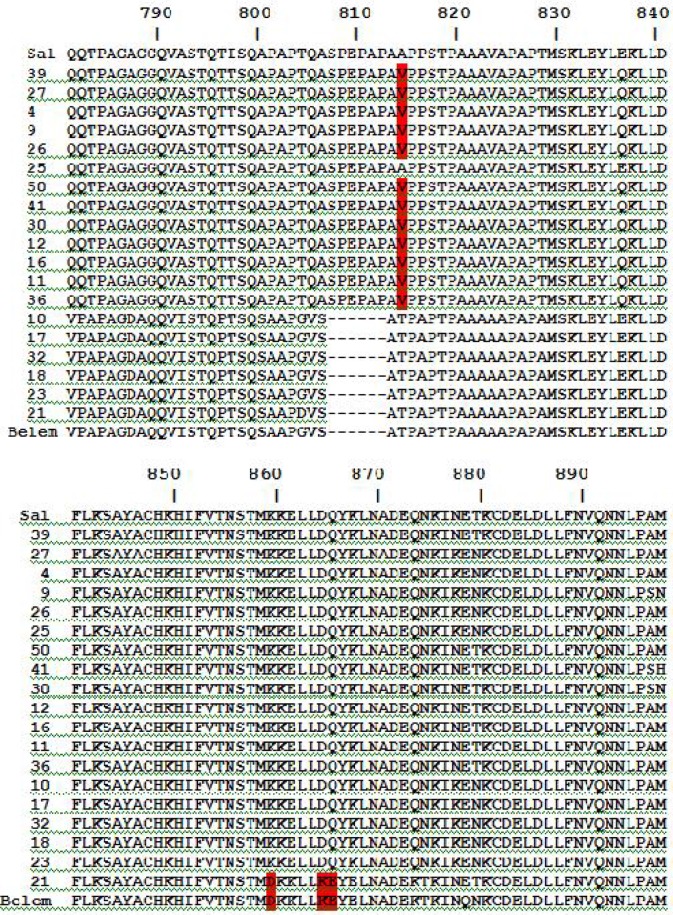
Alignment of decapeptide unites amino acid sequences of MSP-1 gene in the 19 Iranian *Plasmodium vivax* isolates. The sequences were compared with the available sequences of Salvador (AAA29735) and Belem (AAA63427). Dashes represent deletions

Comparison of the amino acid sequences alignment of isolates with Belem and Salvador (Fig. 3), demonstrated that isolates with banding pattern II in SSCP [10, 17, 18, 21, 23, 32], were more similar to Belem strain. In this group there were deletions in 6 amino acid at residues 807-812, which were not seen in others. Although there were similarities in amino acid sequences of isolates in comparison with Belem strain, some differences in some of residues were seen. One of the mutations, which was detected in this group, was replacement of K (lysine) for D (aspartic acid) at residue 860. Isolate 21 showed D, just like Belem. At residue 865, Belem and isolate 21 showed K, while in other isolates of this group it was replaced with D. Residue 866 in Belem and isolate 21 was E (glutamic acid) which was substituted with Q (glutamine) in other members of this group.

Isolates with banding pattern I, III, IV in SSCP [4, 9, 11, 12, 16, 26, 27, 30, 36, 39,41,50], showed significant similarity to Salvador strain with some differences such as replacement of V (valine) for A (alanine) at residue 814 in isolates mentioned above.

**Fig. 4:**
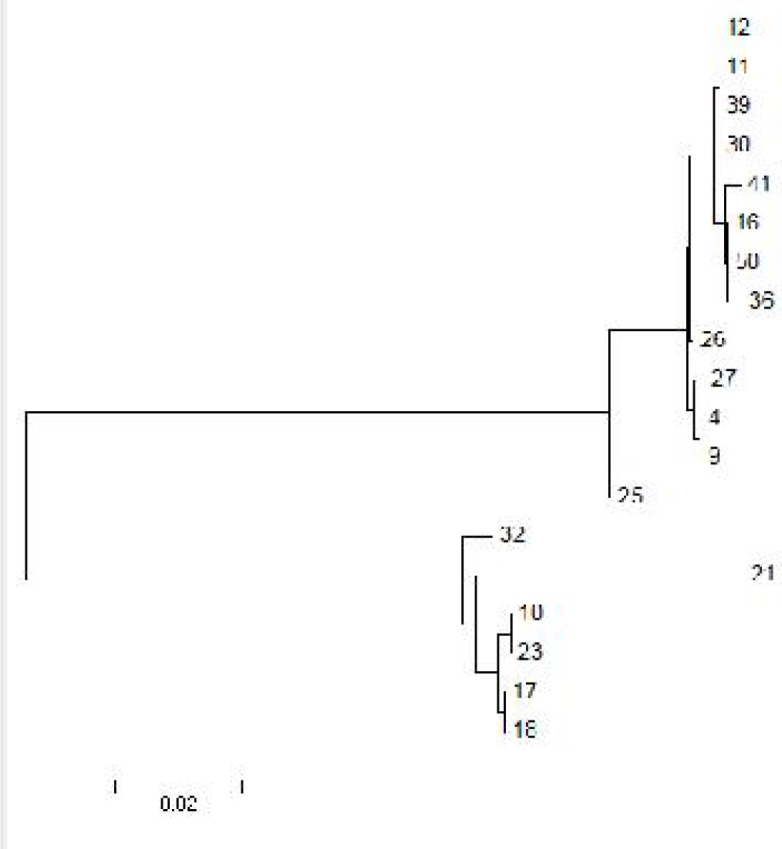
Phylogenetic tree of MSP-1 gene nucleotide sequences. The tree was constructed by using the neighbor-joining method (NJ) with Tamura.3-parameter distance in MEGA version 4.0 software. The numbers on or under the branches indicate the percent of bootstrap values according to 1000 replications

Phylogenetic tree was drawn by MEGA4 software (17), based on the nucleotide sequences of the polymorphic region of *Pv*MSP-1 (Fig. 4).

This analysis separated the isolates into two major heterogenouse groups with different subtype found in each group.

Polymorphic nucleotide sites generated a total of 12 different haplotypes. Nucleotide sequences of different haplotypes in this study have been submitted to GenBank under the accession numbers: JQ292838, JQ292840, JQ343903 - JQ343906, JQ388678 - JQ388683.

## Discussion

In the present study various allelic types were observed in *MSP-1* gene of Iranian *P. vivax* isolates. Genetic variation and polymorphism among these isolates were distinguished due to four different banding pattern groups (I, II, III and IV) determined by SSCP. In electrophoresis of PCR products of these isolates two banding patterns were seen (440 bp and 500 bp). In SSCP single strand DNA is electrophoresed while PCR products electrophoresed when DNA is double strands. So minor variation or deletion can be recognized in SSCP but in PCR it is not available. Incompatibility between the results of PCR and SSCP can be justified by this way.

Sequencing was performed for some samples from each group mentioned above and showed that there was genetic variation in all sequenced isolates which led to allelic diversity in the isolates in south of Iran. Amino acid sequences of 6 out of 19 sequenced isolates were similar to Belem strain. On the other hand, 13 out of 19 sequenced isolates showed more similarity to Salvador strain, which high homology amongst this group was significant while more diversity in amino acid replacement among 6 isolates similar to Belem, was detected.

Comparing the SSCP banding patterns of samples with findings of sequencing indicated that samples with similar banding patterns in SSCP had the most similarity to each other in amino acid sequencing and phylogenetic tree. One of the isolates with banding pattern IV in SSCP had similar amino acid sequence to isolates with pattern III.

Isolates with banding pattern I, III, IV in SSCP, showed some point mutation in different positions in nucleotide sequencing which probably led to various electrophoretic mobility in these isolates with 500 bp used in this study. Our findings were supported by Orita et al. (12) which indicated that minor sequence change could affect on electrophoretic mobility of isolates.

In the study of *Pv*MSP-1polymorphism by sequencing, among 20 isolates of *P. vivax* in 2 regions in Colombia, 5 genetic types of this gene were detected (ST1 to 5) due to identified genetic variation in ICB2 and ICB4 (18). Distinguishable allelic diversity was determined by sequencing in *Pv*MSP-1gene among 68 isolates out of 74 isolates in Thailand from 1995 to 1998 (19). Above studies and ours on genetic variation in *Pv*MSP-1gene, confirmed that there was high genetic diversity in this gene in the world wide.

Genetic variation among isolates from patients in south of Iran, detected by SSCP and sequencing as a typing method, confirmed the diversity, our study were supported by findings of Zakeri et al. who showed genetic variation in the variable block 5 of *MSP-1* among *P. vivax* isolated from patients in the north and south of Iran. Allelic diversity (type 1, type2 and recombinant type 3), was obtained from both regions (20).

A study which was done on isolates from Kolkata in India showed that there was high genetic variation in *Pv*MSP-1gene. Results of sequencing in these isolates showed 35 various and discriminatory allels (21). During our study, isolates detected from Bandar-Jask showed genetic variation which indicated that there were genetic polymorphism in *Pv*MSP-1 in an endemic area and it could influence efficiency of malaria vaccine. It means that it is possible even in small and special region of endemic area all of the isolates which are circulating among patients, do not have the same genetic homology.

In study by Farooq in 2009 in north and northwest of India, 4 allelic types were seen. Using PCR-RFLP divided these 4 allelic types in to 9 sub-allels (22), it seems that using some molecular techniques such as RFLP and SSCP can be useful for detecting genetic diversity when PCR assay is not efficient enough.

Genetic variation in two vaccine candidate genes, *Pvs25* and *Pvs28* in *P. vivax* has been proved by PCR-SSCP. In a study on sextual stage gene (*Pvs25& Pvs28*), 15 haplotypes out of 16 haplotypes were identified by SSCP (23). Our result on asexual stage gene (*Pv*MSP-1) indicated differences among SSCP banding patterns among isolates which showed the effectiveness of this method in determining of polymorphism in malaria vaccine candidate genes.

This study confirmed that polymorphism in MSP-1 gene in the isolates under our study, can affect on their electrophoretic mobility in SSCP. Due to high genotype variation in *Pv*MSP-1 gene, it probably can be possible to detect more banding patterns by increasing the number of samples in Iranian *P. vivax* isolates by SSCP.

## Conclusion

Our study confirms that the SSCP-PCR is a rapid method for detecting polymorphism in *MSP-1* gene in *P. vivax* and the presence of different haplotypes in *MSP-1* gene indicate that several *P. vivax* strains exist in malaria endemic areas of Iran.
